# Assessing the Impact of the Mindfulness-Based Body Scan Technique on Sleep Quality in Multiple Sclerosis Using Objective and Subjective Assessment Tools: Single-Case Study

**DOI:** 10.2196/55408

**Published:** 2024-07-25

**Authors:** Ioannis Iliakis, Maria Anagnostouli, George Chrousos

**Affiliations:** 1 Medical School University of Athens National and Kapodistrian University of Athens Athens Greece; 2 Multiple Sclerosis and Demyelinating Diseases Unit, First Department of Neurology, Medical School Aeginition University Hospital National and Kapodistrian University of Athens Athens Greece; 3 University Research Institute of Maternal and Child Health and Precision Medicine National and Kapodistrian University of Athens Athens Greece

**Keywords:** multiple sclerosis, MS, sleep problems, electronic portable device, EPD, mindfulness-based body scan technique, sleep quality, neurodegenerative disease, quality of life, anxiety, pain, nocturia, assessment tools, single-case study, effectiveness

## Abstract

**Background:**

Multiple sclerosis (MS) is a chronic inflammatory disease affecting the central nervous system, often leading to poor sleep quality and diminished quality of life (QoL) for affected patients. Sleep disturbances in MS do not always correlate linearly with other symptoms such as anxiety, depression, fatigue, or pain. Various approaches, including stress reduction techniques such as mindfulness-based interventions, have been proposed to manage MS-related sleep issues.

**Objective:**

The aim of this study was to evaluate the effects of the mindfulness-based body scan technique on sleep quality and QoL in patients with MS using both subjective (questionnaires) and objective (electronic portable device) measures.

**Methods:**

A single-case study was performed involving a 31-year-old woman diagnosed with relapsing-remitting MS. The patient practiced the mindfulness-based body scan technique daily before bedtime and outcomes were compared to measures evaluated at baseline.

**Results:**

The mindfulness-based body scan intervention demonstrated positive effects on both sleep quality and overall QoL. Biometric data revealed a notable dissociation between daily stress levels and sleep quality during the intervention period. Although self-report instruments indicated significant improvement, potential biases were noted.

**Conclusions:**

While this study is limited to a single patient, the promising outcomes suggest the need for further investigation on a larger scale. These findings underscore the potential benefits of the mindfulness-based body scan technique in managing sleep disturbances and enhancing QoL among patients with MS.

## Introduction

Multiple sclerosis (MS) is an autoimmune disorder that affects the central nervous system (CNS), characterized by neuroinflammation, demyelination, neuronal loss, and gliosis, primarily mediated by T and B lymphocytes [1]. Patients with MS experience significant sleep disturbances, far exceeding those of the general population [2]. Typical pathophysiologic manifestations of MS, such as autonomic nervous system (ANS) and hypothalamic-pituitary-adrenal (HPA) axis dysfunction [3], dysregulation of melatonin secretion [4], CNS inflammation, and oxidative stress, can be linked to primary insomnia [5]. Anxiety and depression, pain, and circadian rhythm disturbance due to disease-modifying treatment (DMT) are secondary factors related to low sleep quality in patients with MS. Other nocturnal manifestations of the condition also contribute to this aspect [6]. The clinical significance of chronic insomnia in people with MS is underscored by multiple studies showing a decreased overall quality of life (QoL) [2,7] and a higher prevalence of anxiety, depression, and daytime fatigue [8-10].

Comorbid anxiety and depression are highly prevalent in patients with chronic illness [11]. Several studies suggest that anxiety can reach a lifetime prevalence of up to 50% for this population [12]. The relationship between stress and sleep is complex, involving various physiological systems. Stress triggers the sympatho-adreno-medullary and HPA systems, thereby inducing cardiovascular, catecholamine, cortisol, adrenocorticotropic hormone, and corticotropin-releasing hormone hyperactivity [13]. Increased cortisol secretion negatively affects sleep [14], while increased ANS activity induces alertness, leading to sleep disturbances such as decreased slow-wave and REM sleep [15]. Conversely, insomnia mirrors the physiological responses seen in stressful situations, such as increased cortisol levels and metabolic activity. Sleep deprivation exacerbates stress by altering hormone levels and increasing appetite. Thus, insomnia and stress form a vicious cycle, with stress leading to sleep disturbances and insomnia further activating the stress response system [16].

Research suggests that mindfulness practices can subjectively enhance sleep quality [17,18]. The efficacy of mindfulness may stem from its ability to stimulate brain regions associated with positive emotions and immunological function, which are both crucial for managing immune system–mediated CNS disorders such as MS. Furthermore, interactions between the prefrontal cortex and the limbic system, which regulate emotional processes, may justify the utility of mindfulness in enhancing sleep quality [19]. One such technique is the mindfulness-based body scan technique, originally introduced by Kabat-Zinn [20], which involves a guided segmental mental scan of the entire body. The selection of this relaxation method for this study stemmed from its capacity to strengthen the mind-body connection and enhance body image [21,22], which are particularly beneficial for individuals facing sensory and mobility challenges such as those with MS. Specifically, this method was chosen owing to its ability to achieve these benefits without requiring physical engagement.

In the current health care landscape, remote medicine is emerging as a potential future standard, particularly for patients facing mobility limitations. Prior studies on remote monitoring in individuals with CNS diseases have primarily focused on the effects of physical exercise programs [18], although a recent investigation contrasted mindfulness training with sleep hygiene practices in patients with MS experiencing chronic insomnia [6]. However, there is a gap in interventions examining the effects of mindfulness sessions before bedtime. Thus, we introduced the use of an electronic portable device (EPD), specifically a smartwatch, to track biometric data related to sleep patterns, QoL, and daily stress levels during a presleep mindfulness intervention period. This approach is innovative as it incorporates a targeted intervention that directly addresses the sleep-related challenges faced by individuals with MS. Understanding how mindfulness affects sleep in patients with MS is key to improving treatment and our overall knowledge of mind-body connections in chronic illnesses.

The objective of this study was to investigate the effects of the mindfulness-based body scan technique prior to bedtime on sleep quality, emotional state, and QoL in a patient with MS. Additionally, we aimed to evaluate the suitability and effectiveness of this technique for patients with MS using both objective (EPD biometrics) and subjective (self-report questionnaires) assessment tools.

## Methods

### Patient

A 31-year-old woman recently diagnosed with relapsing-remitting MS via typical independent neurological and neuroradiological examination presented to the outpatient clinic of the National Center of Rehabilitation (Greece, Athens). Initial symptoms included mild dysarthria, instability, numbness, and impairment of fine movements of the left upper limb. Following diagnosis, the patient underwent DMT with injectable interferon β-1a, resulting in complete resolution of the presenting symptoms.

At the first meeting in the rehabilitation outpatient clinic, the patient reported remission of physical symptoms but complained of ongoing restlessness, anxiety, insomnia, and mild weakness in the left hand. Radiological examination with magnetic resonance imaging revealed multiple brain lesions and a minor lesion in the cervical spinal cord. The clinical examination determined an Expanding Disability Status State score of 1.0. The patient had no significant comorbidities and led an active lifestyle.

### Intervention

The relaxation technique used in this study was adapted from Jon Kabat-Zinn’s mindfulness-based body scan technique [20]. This method involves a guided segmental mental scan of the entire body to achieve relaxation and promote body awareness. During the practice, participants are instructed to lie down with their eyes closed and systematically shift their attention from the toes to the head.

The research period spanned 5 weeks, with the first week dedicated to the preintervention period to collect baseline biometric data using a wearable device (Fitbit Sense 3) and administer 4 self-report questionnaires: the Pittsburgh Sleep Quality Index (PSQI) [23], Depression Anxiety Stress Scale-21 [24], Fatigue Severity Scale [25], and Multiple Sclerosis Quality of Life scale (MSQOL-54) [26]. From the second to the fifth week, the participant engaged in the mindfulness-based body scan technique daily before bedtime. The intervention involved listening to a prerecorded relaxation technique freely translated and narrated by one researcher (II). At the conclusion of the research period, the participant completed the same questionnaires and returned the smartwatch.

### Measures and Data Analysis

The Fitbit Sense 3 measured various biomarkers in real time, including resting heart rate (RHR), heart rate variability (HRV), respiratory rate (RR), body temperature, nighttime sleep, daytime wake period, sleep quality, and stress score. Stress scores were computed using the variables HRV, exertion, and sleep data. Sleep scores considered HRV, bedtime wakefulness, and sleep stages. Data were extracted from the Fitbit online platform and analyzed using SPSS version v26 (IBM Corp, 2020 release). The Mann-Whitney *U* test was used for evaluating the significance of differences in measures between time points and Kendal τ was used for the correlation analysis.

### Ethical Considerations

After assessing the patient’s proficiency with technological equipment and obtaining written informed consent, the Scientific Council of the National Rehabilitation Center of Greece approved the research protocol.

## Results

Analysis of biometric parameters revealed no statistically significant differences between the baseline and intervention time periods for RHR, HRV, RR, body temperature, oxygen saturation, and sleep and stress scores (Table 1).

An intriguing relationship was found between daily stress levels and sleep quality. While this association was evident during the baseline period, it seemed to diminish during the intervention timeframe, suggesting a potential role of the mindfulness-based body scan technique in reducing the correlation between anxiety and sleep quality (Table 2).

**Table 1:** Differences in key variables measured by the Fitbit at baseline and after the intervention.

Variables	Baseline (May 14-20)			Intervention (May 21-June 13)				*U*		*P* value
	Measures, n	Mean (SD)	Median	Measures, n	Mean (SD)	Median				
RHR^a^	7	75 (2.08)	75.00	24	73.58 (2.64)	73.50	56		.18	
HRV^b^	7	36.99 (14.67)	35.51	24	38.90 (11.65)	37.27	69		.48	
Respiratory rate	7	15.54 (1.64)	14.60	24	15.09 (1.55)	14.90	74		.64	
Temperature	7	34.59 (0.91)	34.62	24	34.44 (0.69)	34.63	76		.71	
SpO_2_^c^	5	96.48 (0.88)	96.60	22	96.34 (0.71)	96.40	49		.71	
Sleep score	7	78.43 (7.09)	79.00	24	74.75 (11.01)	74.00	71		.54	
Stress score	7	76.86 (6.44)	77.00	9	78.44 (5.46)	79.00	27.5		.67	

^a^RHR: resting heart rate.

^b^HRV: heart rate variability.

^c^SpO_2_: oxygen saturation.

**Table 2:** Kendall τ correlation coefficients of the Fitbit data^a^.

Variables	RHR^b^	HRV^c^	RR^d^	Body temperature	SpO_2_^e^	Sleep score	Stress score
RHR	1.000	–0.023	0.298	0.158	0.144	0.019	0.157
HRV	0.000	1.000	–0.296	–0.309	0.193	–0.040	0.310
RR	–0.369	–0.683	1.000	0.226	–0.018	0.041	–0.141
Temperature	–0.309	–0.619	0.488	1.000	0.004	0.015	0.028
SpO_2_	0.000	–0.105	0.333	–0.527	1.000	0.274	0.150
Sleep score	0.369	0.683	–0.750	–0.390	–0.527	1.000	0.286
Stress score	0.309	0.619	–0.683	–0.619	–0.316	0.781	1.000

^a^Coefficients in the lower diagonal indicate the values obtained at the first time point (baseline) and coefficients in the upper diagonal indicate values obtained at the second time point (after the mindfulness-based body scan intervention).

^b^RHR: resting heart rate.

^c^HRV: heart rate variability.

^d^RR: respiratory rate.

^e^SpO_2_: oxygen saturation.

Analysis of self-report questionnaires revealed improvements in several parameters reported by the patient. First, the patient experienced an improvement in QoL based on scores from the MSQOL-54 questionnaire, with scores increasing from 70.04 at baseline to 79.35 after the intervention. Second, there was a notable decrease in the severity of fatigue as assessed by the Fatigue Severity Scale, with scores decreasing from 45 to 18 between the first and second time periods.

Third, the patient reported a reduction in perceived stress levels according to the Perceived Stress Scale, with scores decreasing from 28 to 21 over the two time periods. Additionally, there was a slight improvement in sleep quality as measured by the PSQI, with scores increasing from 11 to 12 from the first to the second time period.

## Discussion

### Principal Findings

This case study aimed to investigate the effects of the mindfulness-based body scan technique on a patient with MS when administered before bedtime, focusing on sleep quality, anxiety levels, and overall QoL. The evaluation of parameters involved both objective and subjective measures. The findings can be examined from two perspectives. First, despite the patient reporting improvements in sleep quality according to the PSQI, these subjective improvements were not reflected in the objective biometric data analysis. Second, an interesting observation was the apparent diminishment of the association between the sleep score and stress score over the course of the intervention.

Our study findings echo those of previous research, indicating disparities between subjective self-reported and objective measures of sleep quality. A 2023 study comparing mindfulness training to sleep hygiene in 53 patients with MS experiencing chronic insomnia yielded similar discrepancies between self-reported and objective measurements of sleep quality [6]. Furthermore, a systematic review and meta-analysis conducted in 2019, encompassing 49 studies and 4506 participants, found similar challenges in objectively measuring the effects of mind-body therapies on sleep quantity indices, despite significant improvements in subjective sleep quality and reductions in insomnia severity [27].

To address limitations identified in previous research, our study expanded beyond examining the impact of the relaxation technique solely on sleep. We integrated additional biometric parameters related to the patient’s emotional state, such as HRV, RHR, and the stress score, to explore how the mindfulness-based body scan technique influences sleep quality in individuals with MS. Notably, our EPD data analysis revealed that the daily stress score variable did not significantly affect the sleep score during the intervention, contrasting with the observed correlation during the baseline period. This suggests that the relaxation technique, when performed before night rest, may effectively counteract the adverse effects of daily stressors on sleep quality, thereby enhancing the perception of restful sleep. This finding is important as patients with MS have a significantly higher incidence of anxiety and depressive disorders compared to the general population [28].

Mindfulness-based stress reduction has been found to decrease stress and mood dysregulation, enhance positive goal-setting, and improve emotional capacity [22]. Meta-analyses have confirmed that this technique enhances perceived sleep quality and reduces insomnia severity in diverse populations [27,29].

### Limitations

While existing studies consistently demonstrate the positive effects of relaxation techniques on sleep quality, the translation of these improvements into clear subjective measures presents challenges. Possible reasons for this discrepancy include limitations in sample size, insufficient study duration, and the complexity of the sleep-stress relationship. Moreover, unexplored parameters and inherent biases in questionnaire findings may contribute to inflated perceptions of improvement. Our study does not escape these limitations, as it pertains to only one participant and relies on self-report questionnaires. Thus, while the benefits of relaxation on sleep quality are well-established, addressing these challenges is crucial for accurate assessment and understanding of their impact.

### Conclusion

The mindfulness-based body scan technique demonstrated beneficial effects on both sleep quality and QoL questionnaires in our patient with MS. While we interpret the questionnaire results with caution owing to their subjective nature, the reported feelings of well-being during the intervention are noteworthy. Of particular significance is the observed dissociation between stress levels and sleep quality, suggesting that the mindfulness-based body scan technique may help insulate individuals from anxiety-related thought patterns, thereby promoting a calmer state of mind that is conducive to better sleep. Although these findings are based on a single patient, they provide a foundation for future research. Larger-scale studies involving diverse MS populations are warranted to confirm the validity and generalizability of these results. If replicated, the mindfulness-based body scan approach could offer valuable insights for managing the psychological symptoms of patients with MS and may inform future treatment strategies.

## Abbreviations

ANS: autonomic nervous system
CNS: central nervous system
DMT: disease-modifying treatment
EPD: electronic portable device
HPA: hypothalamic-pituitary-adrenal
HRV: heart rate variability
MS: multiple sclerosis
MSQOL-54: Multiple Sclerosis Quality of Life
PSQI: Pittsburgh sleep quality index
QoL: quality of life
RHR: resting heart rate
RR: respiratory rate
